# Composition and Statistical Analysis of Biophenols in Apulian Italian EVOOs

**DOI:** 10.3390/foods6100090

**Published:** 2017-10-18

**Authors:** Andrea Ragusa, Carla Centonze, Maria Elena Grasso, Maria Francesca Latronico, Pier Francesco Mastrangelo, Francesco Paolo Fanizzi, Michele Maffia

**Affiliations:** 1 Department of Engineering for Innovation, University of Salento, via Monteroni, 73100 Lecce, Italy; 2 CNR Nanotec, Institute of Nanotechnology, via Monteroni, 73100 Lecce, Italy; 3 Department of Biological and Environmental Sciences and Technologies, University of Salento, via Monteroni, 73100 Lecce, Italy; carla_centonze@libero.it (C.C.); nutrizionegrasso@gmail.com (M.E.G.); latronico-francesca@libero.it (M.F.L.); mastrangelo.pf@hotmail.it (P.F.M.); fp.fanizzi@unisalento.it (F.P.F.)

**Keywords:** HPLC, EVOO, biophenols, antioxidants, hydroxytyrosol, oleuropein, multivariate statistical analysis, OPLS-DA

## Abstract

Extra-virgin olive oil (EVOO) is among the basic constituents of the Mediterranean diet. Its nutraceutical properties are due mainly, but not only, to a plethora of molecules with antioxidant activity known as biophenols. In this article, several biophenols were measured in EVOOs from South Apulia, Italy. Hydroxytyrosol, tyrosol and their conjugated structures to elenolic acid in different forms were identified and quantified by high performance liquid chromatography (HPLC) together with lignans, luteolin and α-tocopherol. The concentration of the analyzed metabolites was quite high in all the *cultivars* studied, but it was still possible to discriminate them through multivariate statistical analysis (MVA). Furthermore, principal component analysis (PCA) and orthogonal partial least-squares discriminant analysis (OPLS-DA) were also exploited for determining variances among samples depending on the interval time between harvesting and milling, on the age of the olive trees, and on the area where the olive trees were grown.

## 1. Introduction

The nutritional and health-promoting properties of extra-virgin olive oils (EVOOs) are well-known and they have been extensively studied over recent decades. In particular, it has been demonstrated that phenolic derivatives in olive oils, also known as biophenols, have strong antioxidant activity which, apart from being responsible of the product shelf life, it also confers on EVOOs anti-inflammatory, chemopreventive, and anti-cancer effects, among others, yielding a product with very important nutraceutical properties [1].

**Figure 1:**
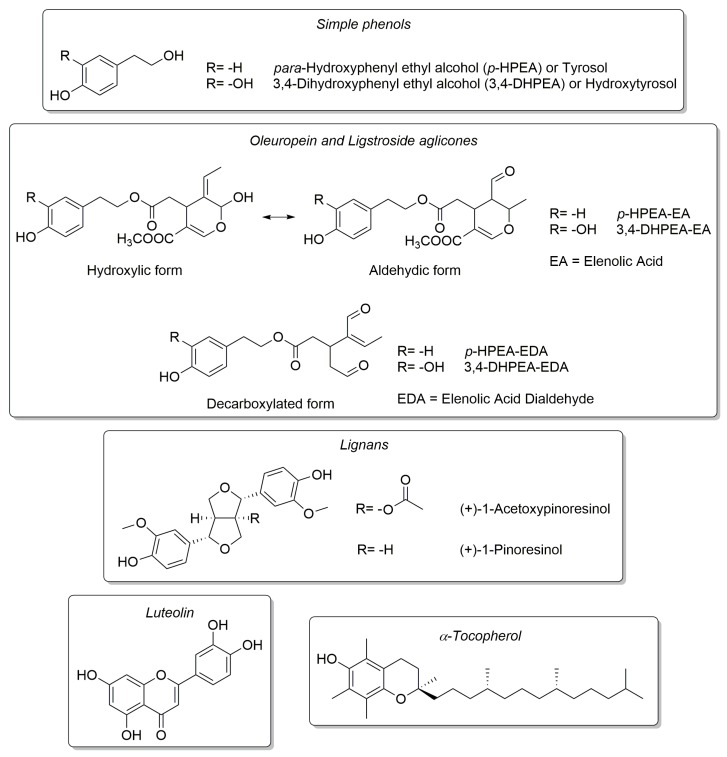
Chemical structures of the antioxidant molecules studied.

Antioxidants in EVOOs can be mainly grouped in two categories depending on the solubility of the phenolic compounds in either organic or aqueous solvents [2]. Lipophilic phenols in EVOOs are mainly constituted by tocopherols, and in particular, their α-homologue, the most biologically active form of vitamin E, which represents over 90% of their total composition [3]. On the other hand, lignans and secoiridoids represent the mayor constituents of hydrophilic phenols in EVOOs, with the latter being only found in plants belonging to the family of *Oleaceae*. Among secoiridoids, *o*-diphenols, such as 3,4-DHPEA and its derivatives 3,4-DHPEA-EDA and 3,4-DHPEA-EA, were reported to have a higher antioxidant activity compared to *p*-HPEA and α-tocopherol (see Figure 1 for names and structures of these compounds) [4]. Nevertheless, all these molecules, together with other minor compounds, such as carotenoids, and thanks to a well-balanced unsaturated-to-saturated fatty acid ratio, contribute to the high quality of EVOOs and its fundamental role in the Mediterranean diet.

EVOOs from Apulia have been already studied in quite significant detail over recent years, also because they represent almost 40% of the total Italian production [5]. However, most of the studies examine *cultivar* from the central area of Apulia and only few investigate the southern part, that is, Salento. Back in 1999, Caponio et al., studied the phenolic composition of Ogliarola and Coratina EVOOs and correlated its variation to different technological variables [6]. They later investigated the influence of decanter working parameters on the quality of the oils [7]. Longobardi and colleagues classified Apulian olive oils based on their chemical composition, i.e., free acidity, peroxide value, spectrophotometric indexes, chlorophyll content, sterol, fatty acid, and triacylglycerol, and on the NMR profile combined with multivariate statistical analysis [8,9]. Gambacorta et al., also inspected the phenolic composition and the antioxidant activity of Collina di Brindisi oils and found out that the maturity degree of olives had a major influence on the phenol composition, followed by the extraction system and the place of growth [10]. Over recent years Fanizzi and colleagues explored, in detail, the use of nuclear magnetic resonance (NMR) and used multivariate statistical analysis (MVA) to investigate various aspects of EVOOs from Apulia. They managed to correlate NMR spectra to genetic profile of the *cultivars*, to the characteristics of the soil where the trees were grown, to the age of the olive trees, as well as the production parameters employed and the weather influence over the years [11,12,13,14]. The NMR-based metabolomics was exploited to generate a comparable data set for fingerprinting olive oil *cultivars* from Apulia, thus allowing identification and traceability of commercial EVOOs [5,15,16,17,18]. They also applied the same technique to EVOOs from Salento, in particular to Ogliarola, Cellina, and blend samples [19]. Nevertheless, a detailed study regarding the phenolic composition in EVOOs from Salento has not been reported so far despite the commercial importance of these products.

We already reported the phenolic profile of several grape types from Salento and we now extend this study, by examining similar antioxidant molecules in local olive oils [20]. In this article, the total amount of biophenols, as well as the concentration of hydroxytyrosol and tyrosol and their derivatives conjugated to elenolic acid, lignans, luteolin, and α-tocopherol were quantified in several monovarietal *cultivars* and blend EVOOs. Furthermore, their concentrations were correlated to the type of *cultivar* and other agronomical parameters, such as the age of the trees and the areas they were located, as well as more technical variables, such as the interval time between harvesting and milling.

## 2. Materials and Methods 

### 2.1. Chemicals

High performance liquid chromatography (HPLC)-grade *orto*-phosphoric acid (85% *v*/*v*) and reference standard of tyrosol, hydroxytyrosol, syringic acid, luteolin, and α-tocopherol were purchased from Sigma-Aldrich (Milan, Italy). HPLC-grade methanol, acetonitrile, isopropanol, and hexane were purchased from J.T. Baker (Deventer, The Netherlands). HPLC-grade water was purchased from Carlo Erba Reagenti (Milan, Italy).

### 2.2. Samples

**Figure 2:**
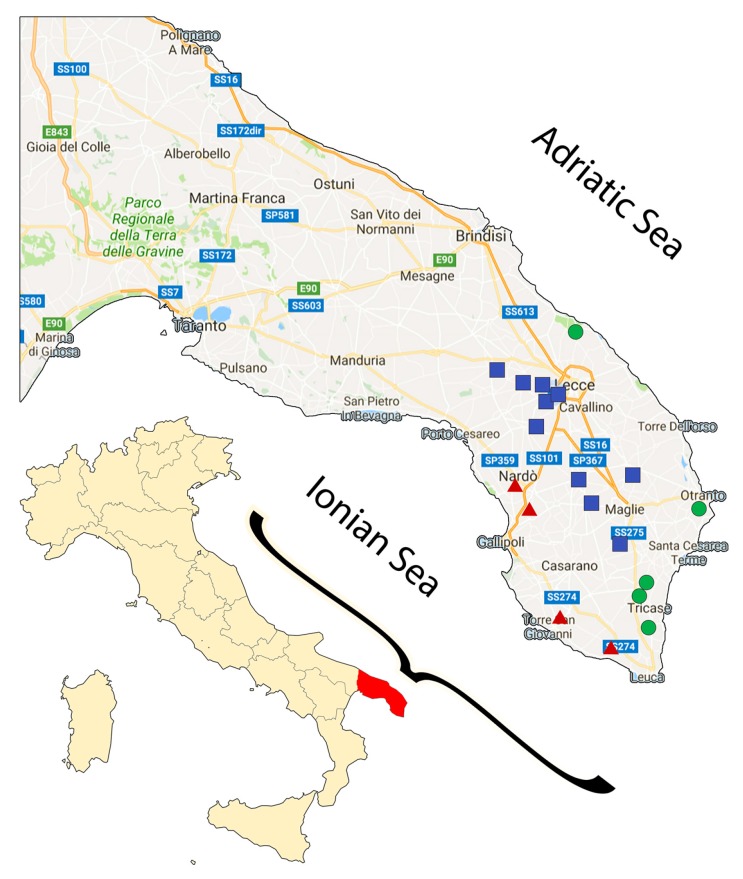
Map of Italy with a zoom on a southern Apulia region (Salento, in red). The colored shapes represent the sites of production of the extra-virgin olive oils (EVOOs) categorized according to their proximity either to the Adriatic (green circles) or Ionian Sea (red triangles), or to neither of them (blue squares).

Analysis was conducted on a total of 68 EVOO samples collected and produced between October and December 2013 by enterprises from Salento, a geographic region at the southern end of the region of Apulia, in South Italy (see Figure 2 for details). All samples were produced from olive trees grown locally and representing typical *cultivars* from Apulia, such as Ogliarola salentina (*n* = 25), Cellina di Nardò (*n* = 20), Leccino (*n* = 4), Frantoio (*n* = 2), Picholine (*n* = 2), and Cima di Melfi (*n* = 1). Furthermore, 14 blend samples, 9 of which composed of mixtures of Ogliarola and Cellina in different ratios (from 30 to 70%), and the other 5 same as the former with also a minor percentage (from 10 to 20%) of either Leccino, Frantoio, or Coratina, were also analyzed. The Protected Designation of Origin (PDO) *cultivars* were certified by the Chamber of Commerce of Lecce (IT), the competent Public Authority Control, while the non-PDO *cultivars* were assessed and certified based on the growers’ declaration. Other technical info and production details were provided by the farmers and are reported in Table 1. When the sum of the number of samples in each category is lower to the total number of samples it means that not all the producers provided the corresponding information. All producers employed the same technical details additionally provided, such as the use of continuous crushers, malaxation at low temperatures (about 25 °C), and two-phases decanters, and as such they were not treated as variables. Samples were stored directly in dark glass bottles soon after production, and were kept as such in a cool, dark place until analyzed. All analyses were completed within 2014.

### 2.3. HPLC Analysis

Separation and identification of phenolic compounds were carried out using an HPLC 1220 Infinity (Agilent Technologies, Palo Alto, CA, USA) equipped with an Eclipse Plus C18 (particle size 5 μm; 4.6 × 250 mm, Agilent) stationary phase column interfaced with a diode array detector (model G1315B DAD system; Agilent).

#### 2.3.1. Analysis of the Phenolic Compounds

Extraction and quantification of the phenolic compounds was carried out following an official methodology from the International Olive Council, see the original document for details and a representative spectrum [22]. Tyrosol and syringic acid in a methanol/water 80/20 (*v*/*v*) solution at a concentration of 0.030 and 0.015 mg/mL, respectively, were used as external standards. Syringic acid in a methanol/water 80/20 (*v*/*v*) solution at a concentration of 0.015 mg/mL was used as internal standard. The concentration of the biomolecules in the analyzed samples was expressed as mg/kg of tyrosol.

Before analysis, 2.0 g of olive oil were put into a 10 mL vial and 1 mL of the internal standard solution was added. The vial was shaken on an orbital shaker (New Brunswick Innova 2050, Eppendorf, Germany) for 30 s and then 5 mL of a methanol/water 80/20 (*v*/*v*) solution were added. The sample was shaken for 1 min and then sonicated for 15 min at room temperature. The sample was centrifuged (Eppendorf 5804R Centrifuge, Eppendorf, Germany) at 5000 rpm for 25 min and an aliquot was extracted from the upper layer and filtered over polyvinylidene fluoride (PVDF) membrane filters with 0.45 μm pore size (VWR International, Milano, Italy). The solution obtained was ready for HPLC analysis.

A ternary gradient system with solvents (A) 0.2% H_3_PO_4_/H_2_O (*v*/*v*), (B) methanol, and (C) acetonitrile was used. The gradient parameters were: 96% (A), 2% (B), 2% (C) at 0 min; 50% (A), 25% (B), 25% (C) at 40 min; 40% (A), 30% (B), 30% (C) at 45 min; 0% (A), 50% (B), 50% (C) at 60 min; 0% (A), 50% (B), 50% (C) at 70 min; 96% (A), 2% (B), 2% (C) at 72 min; 96% (A), 2% (B), 2% (C) at 82 min. The solvent flow was maintained at 1.0 mL/min, the column temperature was set 25 °C, and the ultraviolet-visible (UV-Vis) detection wavelength was set at 280 nm. The ratio of the response factors between syringic acid and tyrosol, *RRF_syr/tyr_*, was calculated before sample analyses to be sure that it was in the range 5.1 ± 0.4. The concentration of the phenol *X* was calculated according to the equation:

*X* (mg/kg) = ((A*_x_*) × 1000 × *RRF_syr/tyr_* × P*_syr_*)/(A*_syr_* × P*_oil_*),

where A*_x_* is the area of the corresponding phenol *X*, P*_syr_* is the weight in mg of the syringic acid utilized as internal standard, A*_syr_* is the area of the peak of syringic acid, and P*_oil_* is the exact weighted amount of oil utilized for the extraction. Obtained retention time for the identified phenols were: hydroxytyrosol, min 10.6; tyrosol, min 15.1; syringic acid, min 20.6; 3,4-DHPEA-EDA, min 31.7; 3,4-DHPEA-EA, min 34.4; *p*-HPEA-EDA, min 36.6; lignans, min 37.8; *p*-HPEA-EA, min 39.8; luteolin, min 40.9; 3,4-DHPEA-EA aldehydic and hydroxylic isomer, min 54.8; *p*-HPEA-EA aldehydic and hydroxylic isomer, min 57.7. The total amount of biophenols was calculated by integrating all the peaks in the spectrum in the range 9–59 min, i.e., those of the identified phenols plus other unidentified. The original raw data are available from the Multilab–Chamber of Commerce of Lecce—upon request at multilab@le.camcom.it.

#### 2.3.2. Analysis of α-Tocopherol

Before analysis, 0.1 g of olive oil was put into a 10 mL vial and 5 mL of methanol were added. The vial was shaken on an orbital shaker for 2 min and then centrifuged at 3000 rpm for 5 min. An aliquot was extracted from the upper layer and filtered over PVDF filters with 0.45 μm pore size (VWR International, Milano, Italy). The solution obtained was ready for HPLC analysis.

A calibration curve was prepared using standard solutions at increasing concentration of α-tocopherol in methanol and recording the corresponding peak area. Fitting of the data was performed through a linear equation with zero intercept (*R*^2^ > 0.99). The concentration in mg/kg of α-tocopherol in the analyzed sample was then calculated through interpolation of the corresponding peak area.

A binary gradient system with solvents (A) 0.5% isopropanol/hexane (*v*/*v*), (B) 10% isopropanol/hexane was used. The gradient parameters were: 100% (A), 0% (B) at 0 min; 100% (A), 0% (B) at 4 min; 60% (A), 40% (B) at 14 min; 40% (A), 60% (B) at 18 min; 100% (A), 0% (B) at 21 min; 100% (A), 0% (B) at 25 min. The solvent flow was maintained at 1.0 mL/min, the column temperature was set 25 °C, and the UV-Vis detection wavelength was set at 288 nm. Obtained retention time was 11.2 min.

### 2.4. Statistical Analysis

The amounts of phenols reported represent the mean values for a specific type of *cultivar*. The reported standard deviation represents the difference among different samples from the same category. When just one sample for a specific type of *cultivar* was available, no standard deviation has been reported. Standard deviation relatively to replicates of the same sample was always <5%. Obtained values were rounded to one decimal place. Statistical analysis was performed using SIMCA 14.1 software (MKS Umetrics, Malmö, Sweden). Principal component analysis (PCA) and orthogonal partial least-squares discriminant analysis (OPLS-DA) were performed using the detected biophenols, their total amount, and α-tocopherol as variables (*n* = 12) while the qualitative information, such as *cultivar*, interval time between harvesting and milling, age of the trees, location of the trees, and PDO certification, as classes according to the data reported in Table 1. Strong outliers were removed from the computation to obtain a better fit. *R*^2^X(cum) and *R*^2^Y(cum) are the cumulative Sum of Squares (SS) of the variation of the X or Y variables, respectively, explained by the extracted components of the model. *Q*^2^(cum) is the cumulative variation of the X and Y variables predicted by the extracted components of the model. *R*^2^X(cum) and *R*^2^Y(cum) are parameters utilized for describing the goodness of the fit and their values are always between 0 and 1, the higher the better. *Q*^2^(cum) is a parameter used to describe the predictive ability of the model and its value is usually between 0 and 1, the higher the better.

## 3. Results and Discussion

The content of several biophenols and their total amount was quantified in several EVOOs from Salento, in Southeast Italy (Figure 2). All EVOOs were prepared from olive trees grown locally from monovarietal species of Ogliarola, Cellina, Leccino, Picholine, Frantoio, and Cima di Melfi. Furthermore, Ogliarola and Cellina were mixed in blend samples, in percentages from 30 to 70%, plus a few samples wherein small amounts of other *cultivar* were added (see Section 2.2 for details). Oils were produced in late 2013, between October and December, and additional agronomical and technical information are reported in Table 1.

**Figure 3:**
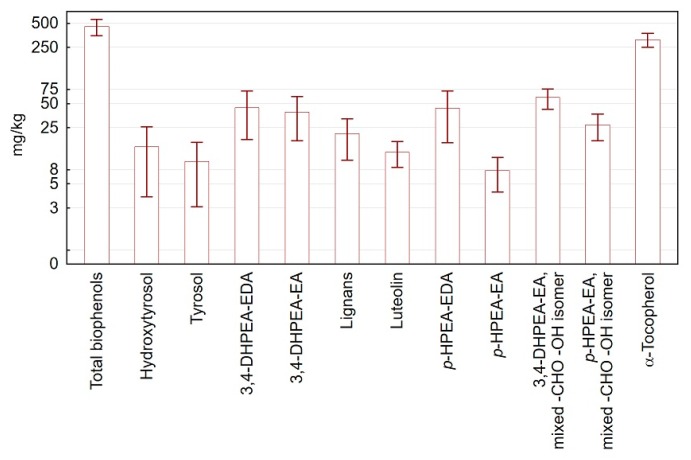
Histograms of the analyzed biophenols averaged for all samples. Bars represent standard deviations. A logarithmic scale was used on the Y axis.

All samples showed significant amounts of the investigated biophenols, as well as very high quantities of α-tocopherol, as shown in Figure 3. *orto*-Diphenols conjugated to the elenolic acid in various forms, i.e., the dialdehydic 3,4-DHPEA-EA, the decarboxylated dialdehydic 3,4-DHPEA-EDA, and 3,4-DHPEA-EA in the mixed aldehydic and hydroxylic form, were found to be the most abundant compounds, followed by their mono-hydroxy homologous (the *p*-HPEA derivatives). Nevertheless, significant amounts of their simple phenols hydroxytyrosol and tyrosol (3,4-DHPEA and *p*-HPEA, respectively) as well as lignans and luteolin were also found.

### 3.1. Analysis by Cultivar

The average total amount of biophenols was about 452 mg/kg, with Frantoio being the *cultivar* with lowest quantity, as detailed in Table 2. On the other hand, Frantoio EVOOs had the highest concentration of hydroxytysosol and tyrosol (about 23 and 20 mg/kg, respectively), and lignans (about 43 mg/kg). Nevertheless, all the other biophenols were present in modest quantities compared to the other *cultivars*. Ogliarola showed quite high concentrations of total biophenols and *p*-HPEA-EDA, all other values being on average. Cellina also had quantities of phenols analogous to average values, with the only exception being the amount of α-tocopherol, the highest among all *cultivars*. As expected, blend samples showed a trend similar to that of Ogliarola and Cellina, being they constituted completely by mixtures in different percentages of those two *cultivars*, or at least for 80% by them also when mixed with other varieties, such as Leccino, Frantoio, or Coratina. The only significant difference was the concentration of α-tocopherol, much lower than that in the pure parent varieties. Small amounts of *p*-HPEA-EDA and *p*-HPEA-EA were detected in Leccino EVOOs, while Picholine had a low concentration of hydroxytyrosol but very high quantities of *p*-HPEA-EA in the aldehydic and hydroxylic form. Finally, Cima di Melfi EVOOs showed quite high concentrations of biophenols compared to average, especially of 3,4-DHPEA-EDA, 3,4-DHPEA-EA, and lignans, while lower quantities of α-tocopherol were detected. However, it must be considered that only two samples each of Frantoio and Picholine and one of Cima di Melfi were analyzed, hence those average values might be quite variable on a broader pattern.

**Figure 4:**
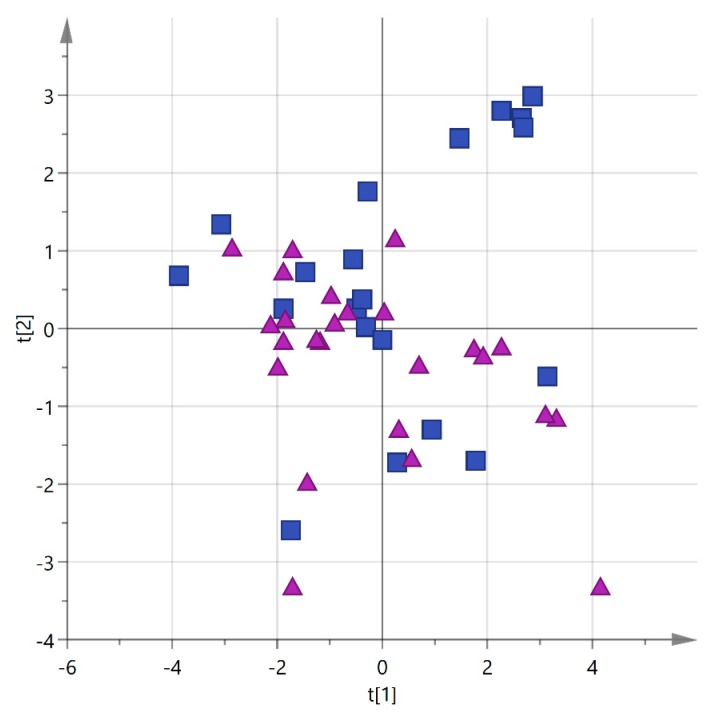
PCA score plot (PC1 vs. PC2) for EVOO samples from Salento categorized by *cultivar* (blue squares = Cellina; purple triangles = Ogliarola).

In order to check the presence of dominant constituents among the *cultivars*, principal component analysis (PCA) was carried out on the Cellina and Ogliarola samples, for a total of 45 observations, using the 10 detected phenols, α-tocopherol, and the total amount of biophenols as variables. Blend samples were initially excluded from the analysis because of their intrinsic variability being mixtures of Cellina and Ogliarola in various ratios and, in some cases, also with other *cultivars*, as already stated. On the other hand, Leccino, Picholine, Frantoio, and Cima di Melfi EVOOs were omitted due to low number of available samples for each category (4, 2, 2, and 1, respectively). The first two components of the model could explain 50.3% of the total population, however the resulting score plot did not show any dominant variable but the samples were quite spread with a high degree of overlap among them, as shown in Figure 4. 

**Figure 5:**
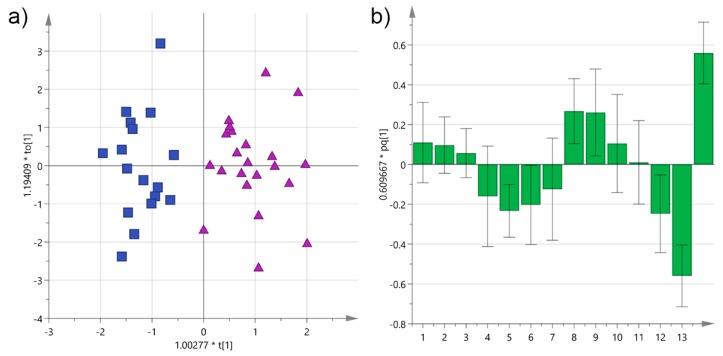
(**a**) Orthogonal partial least-squares discriminant analysis (OPLS-DA) score plot for EVOO samples from Salento categorized by *cultivar* (blue squares = Cellina; purple triangles = Ogliarola); (**b**) OPLS-DA loadings column plot for the first predictive component of the model (variables: 1 = total biophenols; 2 = hydroxytyrosol; 3 = tyrosol; 4 = 3,4-DHPEA-EDA; 5 = 3,4-DHPEA-EA; 6 = lignans; 7 = luteolin; 8 = *p*-HPEA-EDA; 9 = *p*-HPEA-EA; 10 = 3,4-DHPEA-EA in the mixed aldehydic and hydroxylic form; 11 = *p*-HPEA-EA in the mixed aldehydic and hydroxylic form; 12 = α-tocopherol; 13 = Cellina; 14 = Ogliarola).

Much better results were obtained when a supervised multivariate method was used. Partial least-squares discriminant analysis (OPLS-DA) improved significantly separation of the *cultivars*, as shown in Figure 5, with cumulative *R*^2^X and *R*^2^Y of 0.60 and 0.83, respectively, and a respectable *Q*^2^ of 0.66. The two groups were perfectly separated along the mayor component, while the variability among samples is probably responsible for their dispersion along the orthogonal component. According to the loadings, α-tocopherol and 3,4-DHPEA-EA contributed the most to the discrimination of Cellina EVOOs, while *p*-HPEA-EDA and *p*-HPEA-EA to that of Ogliarola samples.

**Figure 6:**
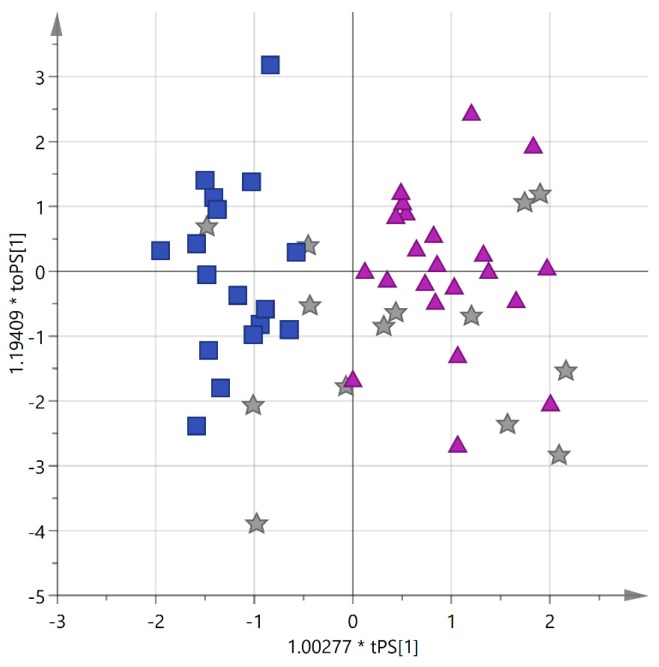
Predicted OPLS-DA score plot for blend EVOO samples from Salento with unknown percentages of Cellina/Ogliarola (blue squares = Cellina; purple triangles = Ogliarola, gray stars = blend predictions).

In order to check the predictive ability of the model, blend EVOOs were used as prediction dataset. As expected, samples did not aggregate in any region of the plot but they spread along PC1 between the two extremes marked by the pure *cultivars*, probably according to the different percentages of Cellina and Ogliarola in their composition, as shown in Figure 6.

### 3.2. Analysis by Interval Time between Harvesting and Milling

**Figure 7:**
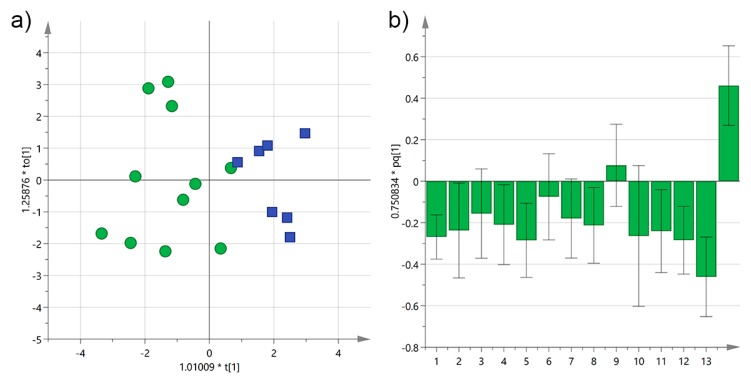
(**a**) OPLS-DA score plot for Cellina EVOO samples from Salento categorized by the interval time between harvesting and milling of the olives (green circles ≤ 12 h; blue squares > 12 h); (**b**) OPLS-DA loadings column plot for the first predictive component of the model (variables: 1 = total biophenols; 2 = hydroxytyrosol; 3 = tyrosol; 4 = 3,4-DHPEA-EDA; 5 = 3,4-DHPEA-EA; 6 = lignans; 7 = luteolin; 8 = *p*-HPEA-EDA; 9 = *p*-HPEA-EA; 10 = 3,4-DHPEA-EA in the mixed aldehydic and hydroxylic form; 11 = *p*-HPEA-EA in the mixed aldehydic and hydroxylic form; 12 = α-tocopherol; 13 ≤ 12 h; 14 > 12 h).

It is well recognized that the amount of antioxidants in EVOOs is influenced, together with other factors, also by the interval time between harvesting and milling. In fact, it has been shown that the shorter this interval time the higher the concentration of biophenols and it is also the reason why, for example, PDO EVOOs are required to be processed by 48 h at the latest after being collected [21]. Samples were grouped in two classes, those which were milled by 12 h from harvesting and those for which that interval time was longer, and OPLS-DA was performed. Initially only Cellina EVOOs were considered to reduce the potential variability introduced by other *cultivars*, and the resulting score scatter plot is reported in Figure 7. 

Discrimination was substantial and it could be observed from the loading plot that most variables contributed significantly to the separation of EVOOs with shorter interval time, especially the total concentration of biophenols, 3,4-DHPEA-EA, and α-tocopherol. On the other hand, only *p*-HPEA-EA appeared to give a slightly positive contribution to samples with an interval time longer than 12 h. However, this result is not surprising because it is known that degradation of antioxidant molecules starts soon after harvesting, and that is why a short interval time between harvesting and milling is a prerequisite for producing high quality EVOOs.

**Figure 8:**
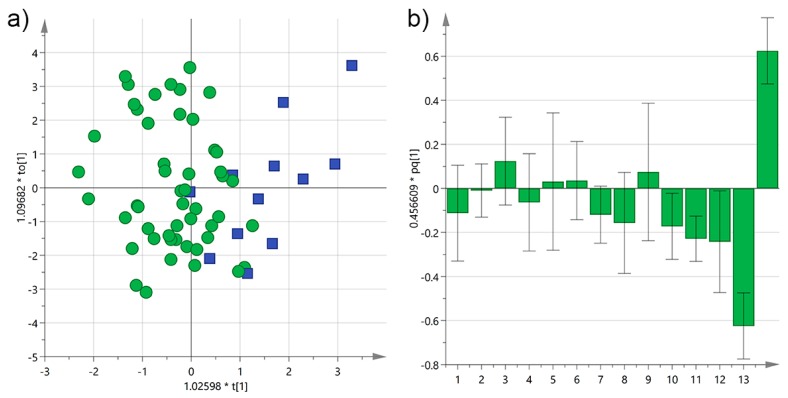
(**a**) OPLS-DA score plot for EVOO samples from Salento categorized by the interval time between harvesting and milling of the olives (green circles ≤ 12 h; blue squares ≥ 12 h); (**b**) OPLS-DA loadings column plot for the first predictive component of the model (variables: 1 = total biophenols; 2 = hydroxytyrosol; 3 = tyrosol; 4 = 3,4-DHPEA-EDA; 5 = 3,4-DHPEA-EA; 6 = lignans; 7 = luteolin; 8 = *p*-HPEA-EDA; 9 = *p*-HPEA-EA; 10 = 3,4-DHPEA-EA in the mixed aldehydic and hydroxylic form; 11 = *p*-HPEA-EA in the mixed aldehydic and hydroxylic form; 12 = α-tocopherol; 13 ≤ 12 h; 14 > 12 h).

Performing the OPLS-DA on all EVOOs yielded a much worse model (*R*^2^X(cum) = 0.38, *R*^2^Y(cum) = 0.45, *Q*^2^X(cum) = 0.28) compared to that obtained with only Cellina samples (*R*^2^X(cum) = 0.56, *R*^2^Y(cum) = 0.73, *Q*^2^X(cum) = 0.54). This was expected given the randomness introduced by other variables, nevertheless a similar trend could be observed in the score scatter plot and in the loading column plot (Figure 8).

### 3.3. Analysis by the Age of the Olive Trees

**Figure 9:**
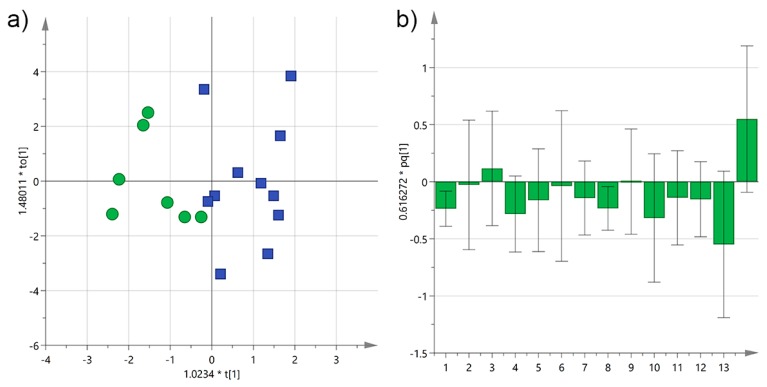
(**a**) OPLS-DA score plot for Cellina EVOO samples from Salento categorized by the age of the olive trees (green circles ≤ 100 years; blue squares > 100 years); (**b**) OPLS-DA loadings column plot for the first predictive component of the model (variables: 1 = total biophenols; 2 = hydroxytyrosol; 3 = tyrosol; 4 = 3,4-DHPEA-EDA; 5 = 3,4-DHPEA-EA; 6 = lignans; 7 = luteolin; 8 = *p*-HPEA-EDA; 9 = *p*-HPEA-EA; 10 = 3,4-DHPEA-EA in the mixed aldehydic and hydroxylic form; 11 = *p*-HPEA-EA in the mixed aldehydic and hydroxylic form; 12 = α-tocopherol; 13 ≤ 100 years; 14 > 100 years).

Salento is well-known for having a high number of secular olive trees, which have recently been regulated and surveyed because of their high historical value [23]. Furthermore, EVOOs from secular olive trees have been already studied and categorized by NMR due to their commercial importance [12]. We now tested if any statistical difference in the amount of biophenols could be detected between secular and younger olive trees. EVOOs were grouped depending on the age of their trees in >100 and <100-year-old classes and OPLS-DA was performed on Cellina samples. A quite good descriptive but not predictive model was obtained, as shown by the correlation coefficients (*R*^2^X(cum) = 0.48, *R*^2^Y(cum) = 0.70, *Q*^2^(cum) = 0.02). According to the loadings, EVOOs from younger trees were significantly discriminated by the total amount of biophenols and *p*-HPEA-EDA, although many other variables also contributed to the separation, as shown in Figure 9. On the other hand, only tyrosol gave a slightly positive contribution to the discrimination of secular trees.

**Figure 10:**
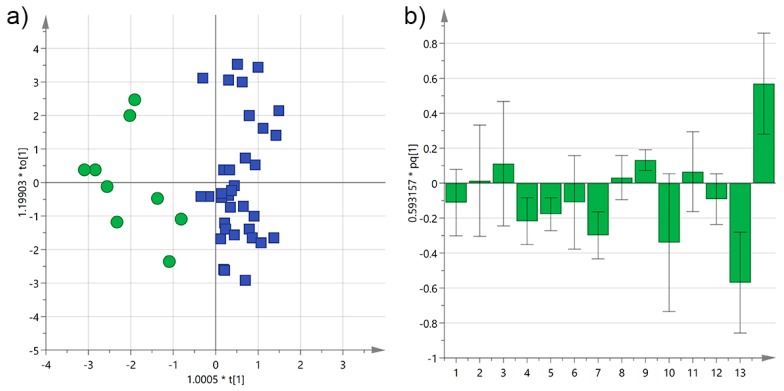
(**a**) OPLS-DA score plot for EVOO samples from Salento categorized by the age of the olive trees (green circles ≤ 100 years; blue squares > 100 years); (**b**) OPLS-DA loadings column plot for the first predictive component of the model (variables: 1 = total biophenols; 2 = hydroxytyrosol; 3 = tyrosol; 4 = 3,4-DHPEA-EDA; 5 = 3,4-DHPEA-EA; 6 = lignans; 7 = luteolin; 8 = *p*-HPEA-EDA; 9 = *p*-HPEA-EA; 10 = 3,4-DHPEA-EA in the mixed aldehydic and hydroxylic form; 11 = *p*-HPEA-EA in the mixed aldehydic and hydroxylic form; 12 = α-tocopherol; 13 ≤ 100 years; 14 > 100 years).

The OPLSA-DA was repeated on all EVOOs yielding, despite the different *cultivars* utilized, a model with very good both descriptive (*R*^2^X(cum) = 0.74, *R*^2^Y(cum) = 0.79) and predictive (*Q*^2^(cum) = 0.65) ability, as shown in Figure 10. The reported loadings again confirmed that younger trees were richer of biophenols compared to secular ones, also emphasizing the role of 3,4-DHPEA derivatives and luteolin, while older ones were discriminated thanks to tyrosol and its derivatives conjugated to elenolic acid.

### 3.4. Analysis by the Cultivation Area

**Figure 11:**
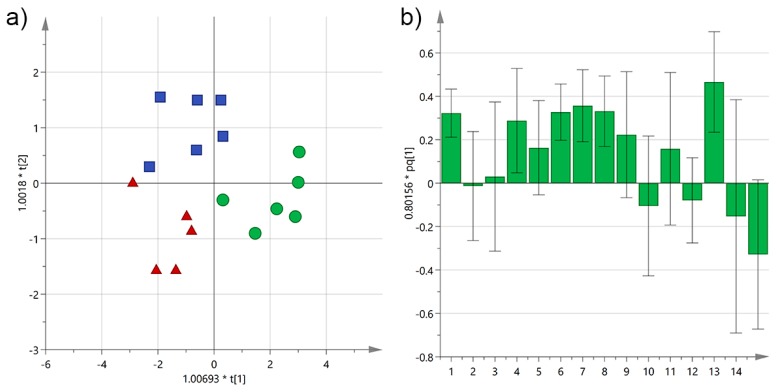
(**a**) OPLS-DA score plot for Cellina EVOO samples from Salento categorized by their proximity either to the Adriatic (green circles) or Ionian Sea (red triangles), or to neither of them (blue squares); (**b**) OPLS-DA loadings column plot for the first predictive component of the model (variables: 1 = total biophenols; 2 = hydroxytyrosol; 3 = tyrosol; 4 = 3,4-DHPEA-EDA; 5 = 3,4-DHPEA-EA; 6 = lignans; 7 = luteolin; 8 = *p*-HPEA-EDA; 9 = *p*-HPEA-EA; 10 = 3,4-DHPEA-EA in the mixed aldehydic and hydroxylic form; 11 = *p*-HPEA-EA in the mixed aldehydic and hydroxylic form; 12 = α-tocopherol; 13 = Adriatic area; 14 = central mainland; 15 = Ionian area).

Climate and agronomical features are very important variables that influence noteworthy the final characteristics of olives and vegetables in general. Gambacorta et al., reported that olive maturity index and technology used had a greater influence with respect to the place of growth on the total amount of phenols [10]. Nevertheless, they found a variability of about 40% depending on the cultivation area. Similarly, we looked for any correlation between the area where the olive trees were grown and the concentration of the analyzed biophenols. OPLS-DA was performed on Cellina EVOOs grouped into 3 categories depending on the proximity of the trees either to the Adriatic or Ionian Sea, or to neither of them if in the central mainland. The EVOOs clustered quite nicely (*R*^2^X(cum) = 0.85, *R*^2^Y(cum) = 0.73) with samples from trees close to the Adriatic Sea being well separated on the positive axis because of most variables, especially luteolin, *p*-HPEA-EDA, lignans, and the total amount of biophenols, as shown in Figure 11. On the other hand, samples from the Ionian area and the central mainland reported both similar negative values along PC1, but were completely separated along PC2 mainly because of *p*-HPEA-EA the former and 3,4-DHPEA-EA in the mixed aldehydic and hydroxylic form the latter.

**Figure 12:**
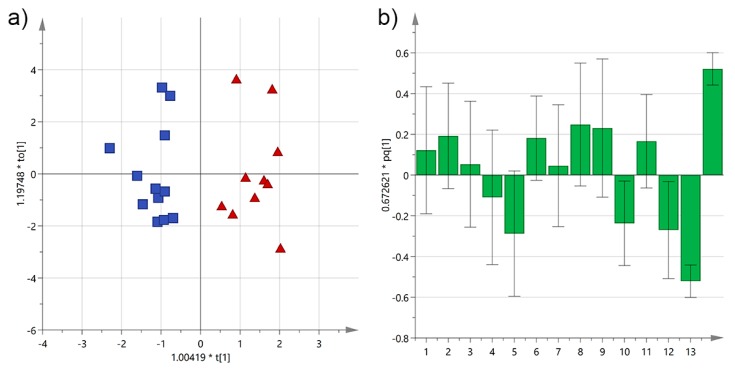
(**a**) OPLS-DA score plot for Ogliarola EVOO samples from Salento categorized by their proximity either to the Ionian Sea (red triangles), or to the central mainland (blue squares); (**b**) OPLS-DA loadings column plot for the first predictive component of the model (variables: 1 = total biophenols; 2 = hydroxytyrosol; 3 = tyrosol; 4 = 3,4-DHPEA-EDA; 5 = 3,4-DHPEA-EA; 6 = lignans; 7 = luteolin; 8 = *p*-HPEA-EDA; 9 = *p*-HPEA-EA; 10 = 3,4-DHPEA-EA in the mixed aldehydic and hydroxylic form; 11 = *p*-HPEA-EA in the mixed aldehydic and hydroxylic form; 12 = α-tocopherol; 13 = central mainland; 14 = Ionian area).

OPLS-DA was also performed on Ogliarola EVOOs, but only samples from the Ionian Sea and the central mainland were considered, being only two those close the Adriatic area. A very good both descriptive (*R*^2^X(cum) = 0.65, *R*^2^Y(cum) = 0.89) and predictive (*Q*^2^(cum) = 0.75) model was obtained, with samples close to the Ionian Sea being richer of *p*-HPEA-EDA and *p*-HPEA-EA, while those from the central mainland of 3,4-DHPEA-EA and α-tocopherol, quite in agreement with Cellina EVOOs from similar cultivation areas (Figure 12).

### 3.5. Analysis by PDO Certification

Protected Designation of Origin (PDO) “Terra d’Otranto” accreditation is a certificate given to EVOOs from the province of Lecce and part of the provinces of Brindisi and Taranto which satisfy determined agronomic and production characteristics which should guarantee healthful properties, among which a high content of antioxidants. Ogliarola and Cellina are the most popular *cultivars* in Salento and they are the mayor components of PDO EVOOs [21,24].

**Figure 13:**
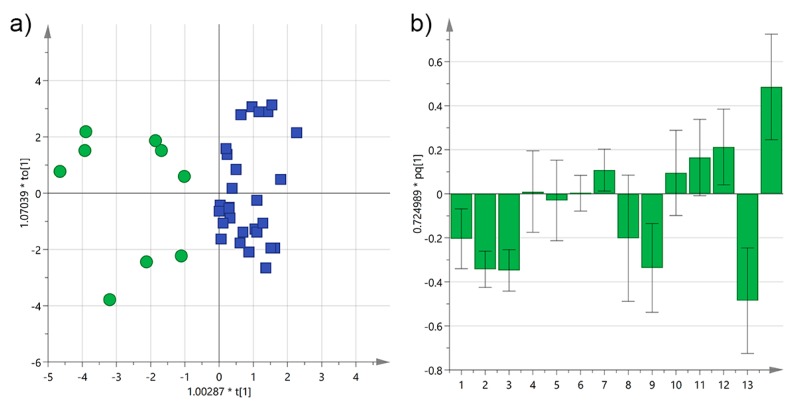
(**a**) OPLS-DA score plot for EVOO samples from Salento categorized by PDO certification (green circles = non-PDO; blue squares = PDO); (**b**) OPLS-DA loadings column plot for the first predictive component of the model (variables: 1 = total biophenols; 2 = hydroxytyrosol; 3 = tyrosol; 4 = 3,4-DHPEA-EDA; 5 = 3,4-DHPEA-EA; 6 = lignans; 7 = luteolin; 8 = *p*-HPEA-EDA; 9 = *p*-HPEA-EA; 10 = 3,4-DHPEA-EA in the mixed aldehydic and hydroxylic form; 11 = *p*-HPEA-EA in the mixed aldehydic and hydroxylic form; 12 = α-tocopherol; 13 = non-PDO; 14 = PDO).

Most of the analyzed EVOOs in this study were PDO certified, however none of the pure *cultivars* studied contained a significant number of both PDO and non-PDO samples to perform a multivariate statistical analysis. Nevertheless, OPLS-DA analysis conducted on all EVOOs could descript (*R*^2^X(cum) = 0.54, *R*^2^Y(cum) = 0.77) and predict (*Q*^2^X(cum) = 0.68) quite satisfactorily the two groups (Figure 13). The PDO samples were separated mainly because of a higher content of α-tocopherol and *p*-HPEA-EA in the mixed aldehydic and hydroxylic form, while smaller phenols, such as tyrosol and hydroxytyrosol, and *p*-HPEA-EA influenced the most the discrimination of samples without certification.

## 4. Conclusions

Quantification of antioxidant molecules in EVOOs is of paramount importance because of their nutraceutical value, even though biophenols represent only minor constituents in olive oil. In this article, we quantified, by HPLC, the total amount of biophenols, several individual biophenols (i.e., tyrosol, hydroxytyrosol, and several more complex derivatives conjugated to elenolic acid in various forms), and α-tocopherol in a variety of pure *cultivars* and blend EVOOs from Salento, in South Apulia. Remarkable differences in the phenolic profile were evident among samples and a high variability was found within the *cultivars*, probably because of the influence of genotyping and other agro-climatic parameters. Nevertheless, all *cultivars* showed significant amounts of biophenols and supervised multivariate statistical analysis could detect differences between Cellina and Ogliarola EVOOs, and predict blend samples. Furthermore, the content of biophenols was correlated to production parameters, such the interval time between harvesting and milling, and to agronomical variables, such as the age of the olive trees and the area they were grown. OPLS-DA showed that shorter interval times lead to an increase in the total concentration of biophenols. Furthermore, it could distinguish between EVOOs from secular and from younger trees, and among samples from different cultivation areas, such as the proximity to the Adriatic or Ionian Sea, or to the central mainland. Eventually, these multivariate statistical analyses, besides yielding valuable information about the characteristics of the studied *cultivars*, could be exploited to improve the already beneficial properties of their EVOOs and to determine a metabolic profile that could be exploited, in combination with other parameters, to guarantee the originality and traceability of these products.

## Figures and Tables

**Table 1:** Technical info and production details about EVOO samples organized by type of *cultivar*. ^1^

*Cultivar*	*n* of Samples	PDO “Terra d’Otranto“ Certification ^2^		Cultivation Area ^3^			Interval Time between Harvesting and Milling (h) ^4^		Age of Olive Trees (year)	
		Yes	No	Ionian	Center	Adriatic	≤12	>12	≤100	>100
Ogliarola salentina	25	19	2	10	13	2	25	-	2	15
Cellina di Nardò	20	17	1	5	8	6	13	7	7	11
Leccino	4	-	-	2	2	-	3	1	1	-
Picholine	2	-	-	2	-	-	2	-	-	-
Frantoio	2	-	-	2	-	-	2	-	-	-
Cima di Melfi	1	-	-	1	-	-	1	-	-	-
Blend	14	8	6	5	5	2	9	4	2	7
*Total*	68	44	9	27	28	10	55	12	12	33

^1^ Extra-virgin olive oils (EVOOs) data were provided by the farmers. When the sum of the number of samples for each category is lower than the total it means that not all the producers provided the corresponding information. ^2^ Protected Designation of Origin (PDO) “Terra d’Otranto” certification is valid only for EVOOs from Ogliarola salentina and Cellina di Nardò *cultivars*, present alone or in combination, in varying percentages and not less than 60%. The remaining 40% can consist of other minor *cultivars* present in the olive grove of the production area [21]. ^3^ Location of the olive trees according to their proximity either to the Ionian or Adriatic Sea, or to neither of them. ^4^ Samples in the “>12” category were milled between 12 and 24 h from harvesting.

**Table 2:** Content of phenols in EVOO samples organized by type of *cultivar*.

*Cultivar*	Total Biophenols	Hydroxytyrosol	Tyrosol	3,4-DHPEA-EDA	3,4-DHPEA-EA	Lignans	Luteolin	*p*-HPEA-EDA	*p*-HPEA-EA	3,4-DHPEA-EA, Mixed Isomer	*p*-HPEA-EA, Mixed Isomer	α-Tocopherol
	Content in mg/kg ^1^											
Ogliarola salentin	468.7 ± 111.4	14.4 ± 8.9	8.2 ± 4.3	39.1 ± 26.7	30.4 ± 18.1	17.8 ± 6.4	12.7 ± 3.8	61.0 ± 32.2	7.9 ± 3.1	61.8 ± 17.4	26.8 ± 8.2	322.5 ± 39.4
Cellina di Nardò	449.1 ± 101.6	14.0 ± 11.6	8.6 ± 6.0	55.1 ± 26.6	44.9 ± 15.8	23.2 ± 11.0	14.8 ± 5.2	34.5 ± 17.4	6.1 ± 2.6	61.9 ± 21.8	25.4 ± 7.4	351.2 ± 52.6
Leccino	379.3 ± 35.8	13.8 ± 20.2	6.8 ± 5.6	44.0 ± 14.7	34.5 ± 11.2	16.5 ± 11.2	11.3 ± 1.3	14.8 ± 2.1	4.3 ± 2.2	63.8 ± 3.8	30.0 ± 7.7	297.8 ± 41.0
Picholine	463.0 ± 62.2	7.0 ± 2.8	6.9 ± 1.4	40.2 ± 22.6	47.5 ± 3.5	9.0 ± 9.9	11.1 ± 0.2	38.0 ± 17.0	4.5 ± 0.7	58.5 ± 2.1	52.3 ± 5.7	265.1 ± 13.1
Frantoio	308.0 ± 83.4	23.0 ± 26.9	20.2 ± 22.6	12.5 ± 2.1	16.0 ± 1.4	43.4 ± 7.1	7.3 ± 2.8	20.1 ± 5.7	5.0 ± 1.4	32.5 ± 7.8	24.5 ± 2.1	189.6 ± 25.1
Cima di Melfi	478.9	4.8	6.1	65.7	62.0	59.3	11.9	37.4	6.7	43.8	19.2	233.9
Blend	462.4 ± 96.8	16.2 ± 10.9	12.2 ± 8.5	40.3 ± 26.6	44.4 ± 28.6	20.9 ± 11.1	10.1 ± 3.4	38.1 ± 21.3	9.9 ± 3.9	54.9 ± 5.1	27.6 ± 12.6	280.4 ± 74.8
*Avg total*	451.6 ± 102.4	14.4 ± 11.2	9.3 ± 6.8	43.9 ± 26.4	38.2 ± 20.6	21.0 ± 11.3	12.5 ± 4.4	43.5 ± 27.6	7.4 ± 3.4	59.3 ± 16.9	27.3 ± 9.7	313.9 ± 61.9

^1^ Values are expressed as mean ± standard deviation relatively to the different samples of EVOO analyzed in each category. Only one sample was available for the *cultivar* Cima di Melfi and no standard deviation has been reported.
